# Off-Patent Generic Medicines vs. Off-Patent Brand Medicines for Six Reference Drugs: A Retrospective Claims Data Study from Five Local Healthcare Units in the Lombardy Region of Italy

**DOI:** 10.1371/journal.pone.0082990

**Published:** 2013-12-18

**Authors:** Giorgio L. Colombo, Enrico Agabiti-Rosei, Alberto Margonato, Claudio Mencacci, Carlo Maurizio Montecucco, Roberto Trevisan

**Affiliations:** 1 Department of Drug Sciences, University of Pavia, Pavia, Italy; 2 S.A.V.E. Studi Analisi Valutazioni Economiche, Milan, Italy; 3 Division of Medicine and Surgery, Spedali Civili, Brescia, Italy; 4 Division of Cardiology, San Raffaele University Hospital, Milan, Italy; 5 Department of Neuroscience, A.O. Fatebenefratelli e Oftalmico, Milan, Italy; 6 Division of Rheumatology, IRCCS Policlinico S Matteo, University of Pavia, Pavia, Italy; 7 Unit of Diabetology, Ospedali Riuniti di Bergamo, Bergamo, Italy; University of Milan, Italy

## Abstract

The scientific documentation supporting the potential clinical and economic benefits of a growing use of off-patent generic drugs in clinical practice seems to be limited in Italy as yet.

**Methods:**

We compared differences in outcomes between off-patent generic drugs and off-patent brand drugs in real clinical practice. The outcomes were: persistence and compliance with therapy, mortality, and other health resources consumption (hospitalizations, specialist examinations, other drugs) and total costs. Retrospective analysis was carried out by using the administrative databases of five Local Healthcare Units (ASLs - Aziende Sanitarie Locali) in the Lombardy Region of Italy. Data from the five ASLs were aggregated through a meta-analysis, which produced an estimate indicator of the mean or percentage difference between the two groups (branded vs. generic) and their respective significance tests. The therapeutic areas and studied drugs were: diabetes: metformin - A10BA02; hypertension: amlodipine - C08CA01; dyslipidemia: simvastatin - C10AA01; psychiatry: sertraline - N06AB06; cardiology: propafenone - C01BC03; osteoporosis: alendronate - M05BA04.

**Results:**

The 5 Local Healthcare Units (ASL) represent a population of 3,847,004 inhabitants. The selected sample included 347,073 patients, or 9.02% of the total ASL population; 67% of the patients were treated with off-patent brand drugs. The average age was 68 years, with no difference between the two groups. After 34 months of observation, compliance and persistence were in favor to generic drugs in all therapeutic areas and statistically significant in the metformin, amlodipine, simvastatin, and sertraline groups. The clinical outcomes (hospitalizations, mortality, and other health costs) show no statistically significant differences between off-patent generic vs. off-patent brand medicines.

**Conclusions:**

Off-patent generic drugs appear to be a therapy option of choice in Italy as well, based on clinical outcomes and economic consequences, both for the National Health Service and patients, considering that the price difference between brand and generic drugs is completely charged on patients.

## Introduction

The appearance of generic drugs on the world pharmaceutical market significantly changed both company strategies and the behaviors of all the stakeholders in health expenditure and in drug prescription [1], [2]. The shift from proprietary product (in-patent), practically produced and marketed exclusively by the innovator pharmaceutical company, to equivalent drug (off-patent*),* potentially produced by an unlimited number of companies, has indeed changed the structure of the reference pharmaceutical market [3]. The presence of the competitive generic drug market in Italy allowed to reduce drug prices by about 40–60% compared with prices before patent expiry [4], [5]. It is well known that generic drugs are medicines made of one or more active ingredients, industrially produced, not protected by a patent or supplementary protection certificate, identified by the international non-proprietary name of the active ingredient or, in its absence, by the drug scientific name, followed by the name of the marketing authorization (MA) holder, bioequivalent to a medicinal product already authorized with the same qualitative and quantitative composition of active ingredients, the same pharmaceutical form, and the same therapeutic indications [6].

Almost half of drug consumption in Italy and about 28% of expenditure is composed of off-patent drugs, although most prescriptions still focus on branded products, while generic (unbranded) drugs are preferred in other European countries. The slow market development that determined these market shares started from the year 2000 and will progressively expand in the next years following new patent expiry. Among off-patent drugs, generic (unbranded) drugs are still a relatively minor market in Italy (about 15% as for quantities and 7% as for expenditure), in comparison with other European countries, where they already represent 60 to 80% of the quantities and 30 to 40% of the expenditure [7]. Important clinical literature supports the full replaceability of off-patent branded with off-patent generic drugs in cardiovascular diseases [8], [9], [10], [11]. In Italy, however, there is only a scarce scientific documentation on the health and social costs and on the outcomes in clinical practice after substituting a branded treatment with generic drugs, which induces a certain mistrust in physicians and patients towards the capabilities of off-patent generic (unbranded) drugs. However, the presence of a relevant market share of off-patent generic (unbranded) drugs is a necessary condition for price competition to unfold among companies after patents expire, with strong reductions and simultaneous benefits for the public health system [12].

In the current contest of decrease and control of public health expenditure, Italian Local Healthcare Units (ASLs, Aziende Sanitarie Locali) have equipped with tools to control expenditure, based on administrative databases (Banca Dati Assistito – BDA) recording and monitoring consumptions and reimbursements to patients by the Italian NHS National Health Service (Servizio Sanitario Nazionale – SSN) [13]. Indeed, administrative databases offer low-cost information (since they are already available) regarding more or less all services provided in a healthcare environment [14], [15]. These sources and their integration are a powerful tool supporting conventional methods used in epidemiological studies.

## Materials and Methods

The purpose of this study was to compare differences in outcomes between off-patent generic medicines and off-patent brand medicines in real clinical practice. The outcomes were as follows: persistence and compliance with therapy, mortality, resource consumption, and other health costs (hospitalizations, specialist examinations, other drugs). The retrospective analysis was developed by using the administrative databases of five Local Healthcare Units (ASLs) (Aziende Sanitarie Locali: Lecco, Bergamo, Pavia, Milano City, and Milano2) in the Lombardy Region, in Italy. We used the following administrative databases: flow of drugs from pharmacies in the area (Farmaceutica Territoriale), database of patients' demographics (Database Anagrafica Assistiti), flow of outpatient specialist examinations (Specialistica Ambulatoriale) and of diagnostic tests and procedures (Diagnostica Strumentale), and flow of hospital discharge forms (Scheda Dimissione Ospedaliera - SDO). In accordance with the Italian privacy law (code concerning the protection of personal data, 30 June 2003, n.196), the analysis was entirely carried out within the single ASLs, which supplied aggregate data following the requested outputs. Data from the 5 ASLs were aggregated through a meta-analysis, which produced an estimate indicator of the mean or percentage difference between the two groups (branded and generic) and their respective significance tests (Table 1). The therapeutic areas and studied drugs were as follows:

**Table 1:** Characteristics of the enrolled patients.

Total patients (5 ASLs)	3,847,004														
		Metformin		Amlodipine		Simvastatin		Sertraline		Propafenone		Alendronate		*Total*	
	ATC	A10BA02	(%)	C08CA01	(%)	C10AA01	(%)	N06AB06	(%)	C01BC03	(%)	M05BA04	(%)	*All patients*	*%*
No. enrolled patient sample	Brand	43,493	57.7%	97,950	84.3%	53,491	58.7%	20,497	57.6%	5,010	61.7%	12,794	61.8%	*233,235*	*67%*
	Generic	31,930	42.3%	18,224	15.7%	37,565	41.3%	15,086	42.4%	3,116	38.3%	7,917	38.2%	*113,838*	*33%*
	Total	75,423	100.0%	116,174	100.0%	91,056	100.0%	35,583	100.0%	8,126	100.0%	20,711	100.0%	*347,073*	*100%*
	Prevalence in enrolled patient sample	1.96%		3.02%		2.37%		0.92%		0.21%		0.54%		*9.02%*	
Distribution per gender of enrolled patients (sample)	Brand (male)	21,451	49.3%	47,652	48.6%	25,259	47.2%	6,186	30.2%	2,580	51.5%	1,238	9.7%	*104,366*	*45%*
	Generic (male)	15,249	47.8%	9,098	49.9%	18,128	48.3%	4,631	30.7%	1,207	38.7%	775	9.8%	*49,088*	*43%*
Mean age of enrolled patients (sample)	Brand	66.31		69.91		69.54		61.13		72.91		73.63		*68.91*	*N.S.*
	Generic	65.61		68.77		68.36		61.73		74.06		73.29		*68.64*	

ASL: Local Healthcare Units. ASLs enrolled: Milano city; Lecco; Bergamo; Pavia; Milano2.

5 ASLs: Milano city; Lecco; Bergamo; Pavia; Milano2.

- diabetes: metformin - A10BA02
- hypertension: amlodipine - C08CA01
- dyslipidemia: simvastatin - C10AA01
- psychiatry: sertraline - N06AB06
- cardiology: propafenone - C01BC03
- osteoporosis: alendronate - M05BA04

The studied drugs for our analysis were selected based on the consideration that, at the time of data extraction (year 2008), at least 10% of the prescription volumes in the considered therapeutic area were generic drugs.

### Patient selection criteria

We included all patients who received, at least one delivered prescription of one of the study drugs, in the above mentioned areas between January 2008 and December 2008. The date of first drug delivery is considered as the index date. Patients were observed for a period of 34 months starting from the index date (including index date). In order to consider only new patients, we applied a 12-month wash-out in which patients did not have a delivered prescription of the studied drugs. We also excluded patients with only one prescription of the studied drugs (sporadic patients) and patients who received a prescription of both generic and branded off-label drugs in the observation period. Some cohorts were defined in order to avoid any biases induced by the presence of multiple diseases, which may have a strong impact on the variability of the primary objectives. For the psychiatry cohort, we only considered sertraline 50 mg or sertraline 100 mg in order to exclude patients with anxiety. In the cohort treated with propafenone, we excluded patients with at least one delivered prescription of an extended release formulation, because the generic extended release formulation is not available yet. Finally, for the alendronate cohort, we excluded patients with a delivered prescription of systemic corticosteroids in the two months prior to the index date, in order not to consider patients with corticosteroid induced osteoporosis.

### Outcome indicators

Persistence was the period of therapy days between the first dispensing and therapy interruption. Persistence is calculated as a continuous variable, in terms of number of therapy days for which the therapy is available, without interruption. The total number of therapy days was analyzed by means of the Defined Daily Dose (DDD) [16]. Intervals, called “maximum allowed gaps” (GAP), were defined according to the kind of analyzed therapy; the maximum time intervals between two deliveries were defined, in order to consider therapy interruptions (Table 2).

**Table 2:** Analysis of persistence in therapy: continuation of therapy (DDD duration) for the recommended period of time.

	GAP	Type	No.	Min	ASL associated with min	Max	ASL associated with max	Mean Diff.	SD	CI 95% Low. Lim.	CI 95% Upp. Lim.	p-value
Metformin	90 days	Branded	6410	305.7	Milano city	435.5	Lecco	67.23	6.56	54.38	80.08	<0.0001
A10BA02		Generic	7688	254.3	Lecco	508.4	Melegnano					
Amlodipine	90 days	Branded	11435	367.8	Milano city	441.9	Bergamo	78.69	6.74	65.47	91.91	<0.0001
C08CA01		Generic	5101	441.6	Milano city	535.5	Bergamo					
Simvastatin	90 days	Branded	6355	281.15	Milano city	365.2	Melegnano	79.79	5.89	68.25	91.33	<0.0001
C10AA01		Generic	10133	348.7	Milano city	428.1	Melegnano					
Sertraline	30 days	Branded	3822	144.9	Milano city	183.1	Melegnano	23.48	5.21	13.28	33.68	<0.0001
N06AB06		Generic	3176	119.2	Lecco	231.7	Melegnano					
Propafenone	30 days	Branded	805	197.1	Milano city	291.9	Bergamo	9.07	21.08	−32.24	50.39	N.S.
C01BC03		Generic	328	137.5	Lecco	274.0	Bergamo					
Alendronate	60 days	Branded	1821	281.4	Pavia	369.1	Melegnano	41.07	11.66	18.22	63.91	0.0004
M05BA04		Generic	1605	328.1	Milano city	401.1	Lecco					

DDD: Defined Daily Dose.

ASL: Local Healthcare Units. ASLs enrolled: Milano city; Lecco; Bergamo; Pavia; Milano2.

GAP: The maximum time intervals between two deliveries; “maximum allowed gaps”.

N.S. not statistically significant.

Compliance to therapy was calculated by means of the Medical Possession Ratio (MPR). MPR was defined as the ratio between the number of packs in the period of persistence multiplied by the number of DDDs per pack, divided by the total days until change of therapy (i.e., persistence) (Table 3).

**Table 3:** Analysis of patients' compliance for persistent patients (MPR - Medical Possession Ratio).

	Type	No.	Min	ASL associated with min	Max	ASL associated with max	Mean Diff.	SD	CI 95% Low. Lim.	CI 95% Upp. Lim.	p-value
Metformin	Branded	6410	0.47	Milano city	0.55	Lecco	0.03	0.01	0.02	0.04	<0.0001
A10BA02	Generic	7688	0.46	Lecco	0.58	Melegnano					
Amlodipine	Branded	11435	0.75	Milano city	0.84	Melegnano	0.04	0.01	0.04	0.05	<0.0001
C08CA01	Generic	5101	0.79	Milano city	0.87	Melegnano					
Simvastatin	Branded	6355	0.42	Milano city	0.48	Melegnano e Lecco	0.03	0.01	0.02	0.04	<0.0001
C10AA01	Generic	10133	0.45	Milano city	0.50	Melegnano					
Sertraline	Branded	3822	0.63	Milano city	0.68	Bergamo e Melegnano	0.03	0.01	0.01	0.04	0.0003
N06AB06	Generic	3176	0.63	Lecco	0.72	Melegnano					
Propafenone	Branded	805	0.62	Milano city	0.72	Melegnano	0.04	0.02	−0.01	0.08	N.S.
C01BC03	Generic	328	0.65	Lecco	0.83	Melegnano					
Alendronate	Branded	1821	0.68	Lecco e Pavia	0.73	Melegnano	0.01	0.01	−0.01	0.03	N.S.
M05BA04	Generic	1605	0.69	Milano city	0.74	Bergamo					

ASL: Local Healthcare Units. ASLs enrolled: Milano city; Lecco; Bergamo; Pavia; Milano2.

DDD: Defined Daily Dose.

N.S. not statistically significant.

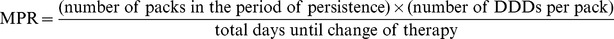

Several others outcomes were analyzed in order to compare the use of generic drugs with the use of brand drugs. Data on hospitalization rates (i.e., number of hospital discharges) was analyzed considering patients that were persistent over a period of at least 6 months (1 year for patients taking simvastatin or propafenone). We considered hospital discharges within the period of therapy and all-cause hospitalizations. Like hospitalization rates, the average number of specialist examinations was calculated considering persistent patients over the period of therapy and all-cause specialist visits. Data on patient mortality was calculated considering persistent patients over the same period considered for hospitalizations and, in this case as well, we considered all-cause mortality. Death was considered from the index date until the end of follow-up. Regarding costs analyses, we defined a total cost for patients, stratified by patients treated with generic drug and treated with branded drug, considering the sum of total costs of all prescriptions, all-cause hospitalizations, all-cause outpatient services, and all-cause exams during the one-year persistence period. Another cost-analysis was performed on a scenario including only prescriptions related to the disease and only hospitalizations related to the disease, with all-cause data for outpatient services and exams. All costs was evaluated from the Italian NHS (National Health Service) point of view.

### Statistical analysis

The qualitative variables were shown with the help of descriptive statistical methods such as frequencies and percentages. The patients' demographic profile for each therapeutic area was expressed in terms of gender (number and percentage), and age classes (number and percentage) at index date. Prescriptions were stratified by therapeutic area, molecule, and number of packs (total number of packs, number of patients, average number of packs per patient, and average number of DDDs per patient). The total treatment cost (hospitalizations, outpatient clinic procedures, other drugs) were stratified by therapeutic area and molecule (in euros). The quantitative variables were described in terms of means, minimum and maximum values, and standard deviation.

The meta-analysis was aimed at combining summary statistics from various studies, all with the same purpose, in order to obtain a mean effect with a higher associated statistical potency. This allowed to find a statistically significant result which single studies could not obtain. In general, retrieving all information sources, both published and unpublished, in order to avoid a selection bias, assessing their quality, and defining a common outcome for all analyses are the biggest problems in using this statistical method [17], [18]. Since our meta-analysis refers to different but well-defined clinical conditions studied in five different Lombardy Local Healthcare Unit (ASLs: Milano City, Bergamo, Pavia, Milano2, Lecco), there was no need to carry out a bibliographic research. Moreover, no problem was encountered in the search for comparable results of the single ASL analyses, since the same analytical procedures of extraction were used. Finally, the single analyses showed statistics of different outcomes, separated by branded or generic drug; for this reason, we chose to create their difference, in order to obtain a single reference measure for each analyzed indicator. Consequently, the meta-analysis refers to differences in means or in proportions, depending on the analyzed outcome. The associated standard deviations were obtained through: 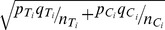 for the difference between proportions and
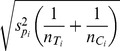
, where 
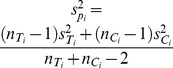
, for the difference between means,

After defining the summary results on which the meta-analysis should be developed, it was necessary to determine how to weigh statistics in the different studies, i.e., choose the model of analysis to be used: fixed effects or random effects. The first model assumes that there are no intra-study variations and that the real researched effect is shared by all studies; weights are therefore simply assigned based on the variability found in the cohort used in the study. The second model, on the other hand, admits the possibility that the researched effect differs from one study to another, assigning weights that take into account the variance both within the studies and between one study and another.

We decided to use the simpler fixed effects model, because the data derived from analyses carried out in the same conditions and with the same objectives. Consequently, the differences in means or proportions will be tied taking into account the associated variances. More formally, the weight *W_i_* is defined as 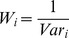, where *Var_i_* is intra-study variance, whereas the general weighted effect 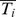 is defined as 
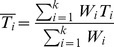
.

Finally, it is possible to obtain the confidence interval for 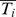, since the mean square combined deviation of the effect is 
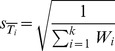
, and calculate the total z-statistics, as 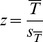. Thus, the test relating to the studied mean effect (be it difference in means or in proportions) will verify if there is a significant deviation of such effect to the zero value. In other words, the test will tell us if there are significant differences between branded and generic drugs, for each analyzed outcome [17], [18].

## Results

### Characteristics of the enrolled patients

**Figure 1:**
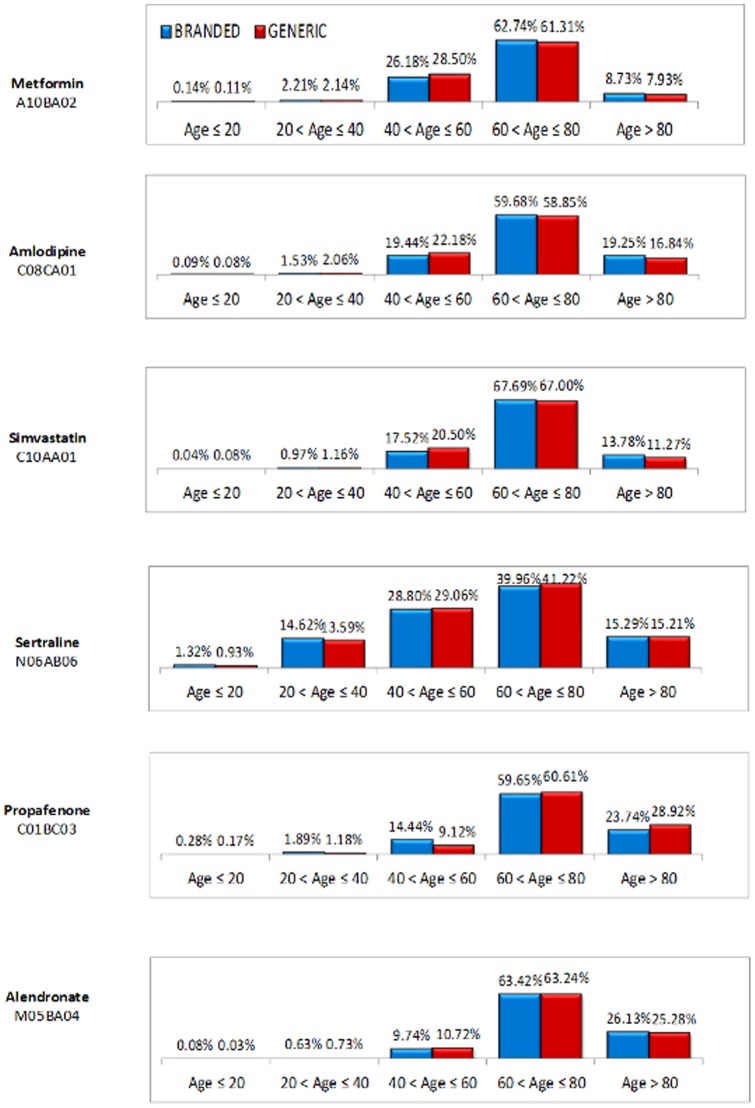
Distribution of the sample per age group and studied drug.

The 5 Local Healthcare Units (ASLs) involved in the survey represent a population of 3,847,004. The selected sample included 347,073 patients, or 9.02% of the ASLs population; 67% of the patients were treated with off-patent branded drugs. The average age was 68 years, with no difference between the two groups; the largest group in terms of percentages included patients aged 60 to 80 years. Table 1 and Figure 1 show the characteristics of the analyzed patients. No statistically significant differences are found in two groups of studied patients, both as to the considered age groups and as to gender.

### Persistence, compliance to therapy and outcome indicators

The number of days in which the patients were persistent varied in average between 267 and 376 in the two groups. It is interesting to notice (Table 2) that patients have a better persistence with generic drugs (about 49 days), with statistically significant differences. Patients' compliance varied between 61% and 69% on average (Table 3), with significant differences in favor of the groups treated with generics as well, except in the propafenone (C01BC03) and alendronate (M05BA04) groups but, in this case, the difference was not significant.

Data on hospitalizations and specialist examinations of persistent patients only did not show differences between the two groups (Tables 4 and 5). Differences in mortality are in favor of the generic group, with the exception of patients treated with propafenone (C01BC03) (Table 6).

**Table 4:** Hospitalizations in persistent patients (mean number of hospitalizations).

	Type	No.	Min	ASL associated with min	Max	ASL associated with max	Mean Diff.	SD	CI 95% Low. Lim.	CI 95% Upp. Lim.	p-value
Metformin	Branded	1408	1.13	Lecco	1.33	Pavia	0.03	0.02	−0.01	0.07	N.S.
A10BA02	Generic	1490	1.20	Bergamo	1.28	Lecco e Pavia					
Amlodipine	Branded	3832	1.26	Melegnano	1.31	Pavia e Milano city	−0.02	0.03	−0.07	0.04	N.S.
C08CA01	Generic	677	1.18	Lecco	1.32	Pavia e Bergamo					
Simvastatin	Branded	1657	1.20	Lecco	1.28	Milano city	−0.01	0.02	−0.05	0.03	N.S.
C10AA01	Generic	1391	1.21	Pavia	1.29	Lecco e Melegnano					
Sertraline	Branded	310	1.14	Pavia	1.26	Lecco	−0.04	0.04	−0.13	0.04	N.S.
N06AB06	Generic	313	1.06	Lecco	1.47	Pavia					
Propafenone	Branded	127	1.00	Pavia	1.40	Melegnano	−0.03	0.06	−0.14	0.08	N.S.
C01BC03	Generic	101	1.00	Melegnano	1.50	Pavia					
Alendronate	Branded	310	1.07	Lecco	1.30	Milano city	0.002	0.04	−0.08	0.08	N.S.
M05BA04	Generic	234	1.12	Melegnano	1.31	Bergamo					

ASL: Local Healthcare Units. ASLs enrolled: Milano city; Lecco; Bergamo; Pavia; Milano2.

N.S. not statistically significant.

**Table 5:** Specialist examinations in persistent patients (mean number of specialist examinations).

	Type	No.	Min	ASL associated with min	Max	ASL associated with max	Mean Diff.	SD	CI 95% Low. Lim.	CI 95% Upp. Lim.	p-value
Metformin	Branded	11645	2.50	Bergamo	3.10	Melegnano	0.02	0.03	−0.04	0.08	N.S.
A10BA02	Generic	11585	2.54	Bergamo	3.00	Milano city					
Amlodipine	Branded	21350	2.61	Pavia	3.24	Lecco	−0.05	0.06	−0.17	0.06	N.S.
C08CA01	Generic	3369	2.65	Milano city	2.95	Melegnano					
Simvastatin	Branded	10554	2.55	Pavia	3.30	Lecco	−0.01	0.04	−0.11	0.07	N.S.
C10AA01	Generic	8988	2.66	Pavia	3.00	Melegnano					
Sertraline	Branded	1868	2.81	Pavia	3.64	Lecco	0.15	0.09	−0.03	0.34	N.S.
N06AB06	Generic	2261	2.86	Lecco	3.59	Melegnano					
Propafenone	Branded	1002	3.62	Pavia	6.39	Bergamo	−0.1	0.27	−0.63	0.44	N.S.
C01BC03	Generic	776	4.79	Pavia	6.12	Lecco					
Alendronate	Branded	2607	2.56	Pavia	3.21	Milano city	−0.02	0.09	−0.19	0.15	N.S.
M05BA04	Generic	1976	2.62	Pavia	3.14	Bergamo e Melegnano					

ASL: Local Healthcare Units. ASLs enrolled: Milano city; Lecco; Bergamo; Pavia; Milano2.

N.S. not statistically significant**.**

**Table 6:** Percentage of deceased among persistent patients.

		Min	ASL associated with min	Max	ASL associated with max	Mean Diff. (in %)	p-value
Metformin	Branded	0.0485	Pavia	0.0906	Lecco	−0.23	N.S.
A10BA02	Generic	0.0437	Pavia	0.0878	Lecco		
Amlodipine	Branded	0.0699	Pavia	0.1191	Melegnano	−1.93	<0.0001
C08CA01	Generic	0.0613	Pavia	0.0975	Bergamo		
Simvastatin	Branded	0.0517	Pavia	0.0850	Bergamo	−1.35	<0.0001
C10AA01	Generic	0.0492	Pavia	0.0697	Melegnano		
Sertraline	Branded	0.0900	Pavia	0.1603	Lecco	−1.15	N.S.
N06AB06	Generic	0.0803	Pavia	0.1161	Melegnano		
Propafenone	Branded	0.0543	Pavia	0.0895	Milano city	3.09	0.0119
C01BC03	Generic	0.0893	Milano city	0.1797	Melegnano		
Alendronate	Branded	0.6940	Pavia	0.1200	Bergamo	−1.06	N.S.
M05BA04	Generic	0.0689	Milano city	0.1034	Bergamo		

ASL: Local Healthcare Units. ASLs enrolled: Milano city; Lecco; Bergamo; Pavia; Milano2.

N.S. not statistically significant.

Table 7 shows the mean health costs for persistent patients only in a year of observation, and the relative differences among the groups. The mean annual cost in the branded group is 784 Euro vs. 739 Euro in the generic group. It is important to note, however, that cost differences were not significant, with the exception of the propafenone group (C01BC03). These differences in costs between the two groups were not statistically significant.

**Table 7:** Mean yearly health costs per patient among persistent patients.

	Type	No.	Cost per specialist examinations	Cost per others drugs	Cost per Hospitalizations	Total annual cost	Mean Diff. (generic - branded)
Metformin	Branded	13473	€ 393.40	€ 208.83	€ 157.83	€ 760.06	€ 12.75
A10BA02	Generic	12950	€ 391.05	€ 184.79	€ 196.97	€ 772.81	
Amlodipine	Branded	33965	€ 438.52	€ 315.50	€ 177.98	€ 932.00	−€ 16.27
C08CA01	Generic	4868	€ 402.40	€ 286.99	€ 226.35	€ 915.73	
Simvastatin	Branded	13679	€ 430.53	€ 180.35	€ 218.18	€ 829.05	−€ 84.25
C10AA01	Generic	11642	€ 411.39	€ 153.49	€ 179.93	€ 744.81	
Sertraline	Branded	1801	€ 392.32	€ 260.15	€ 37.56	€ 690.03	−€ 44.19
N06AB06	Generic	1987	€ 388.15	€ 234.48	€ 23.21	€ 645.85	
Propafenone	Branded	1408	€ 503.43	€ 201.89	€ 23.48	€ 728.80	−€ 128.16
C01BC03	Generic	1002	€ 428.70	€ 149.48	€ 22.46	€ 600.64	
Alendronate	Branded	3008	€ 398.53	€ 336.81	€ 32.64	€ 767.99	−€ 13.30
M05BA04	Generic	2226	€ 431.12	€ 294.31	€ 29.25	€ 754.68	

## Discussion

Although off-patent generic drugs were introduced in therapy many years ago and now encompass over 50% to 70% of prescriptions in the most advanced countries from the healthcare point of view, they are still regarded with a sort of skepticism in Italy, both by specialists and general practitioners, and by the public. This puts Italy in one of the last positions, among European nations, in the use of this clinical tool, although according to the WHO “All health systems, everywhere, could make better use of resources, whether through better procurement practices, broader use of generic products…” [19]. In order to reassure prescribers and to test its policy on generics, the FDA conducted an important research [20] which re-assessed all studies on bioequivalence (2070) carried out in a period of 12 years (1996–2007) to obtain approval of generics for oral use, both with immediate release (1788 studies) and with modified release (282 studies). In 97.6% of the studies, the generic drug AUC (*area under the curve*) differed by less than 10% from that of the innovator product [20]. Moreover, a 2008 review [9] assessed the clinical equivalence of generic and branded drugs in cardiovascular disease, by evaluating 47 papers published in recent literature on nine subclasses of cardiovascular drugs (betablockers, ACE-inhibitors, calcium antagonists, antiplatelet drugs, and statins), most of which (81%) were randomized and controlled clinical studies. The results of this important review highlight that, from the point of view of clinical effectiveness, therapeutic effects, and safety, there are no measurable differences between the original (branded) drugs and generic equivalent drugs [9]. In the treatment of the central nervous system, as well, antidepressant therapy with a generic SSRI or SNRI does not appear to be associated with a higher probability of therapy interruption and is linked with a significant reduction of health costs [21]. Furthermore, generic drugs, with their low purchase price, completely or almost completely reimbursed by the NHS, may favor better adherence to treatment, because it is known that patients who need to contribute to the drug expenditure, even if partially, may not adhere to therapy in an optimal way [22], [23], [24], [25]. In a cohort of 39,714 Dutch patients who had started antihypertensive therapy, 463 patients were picked out who had later switched to generic drugs, and 595 control patients: 13.6% of patients were found non-adherent in the first group, and 18.7% in the control group, with the limitation of a time horizon of 6 months of observation [26].

In spite of this relevant literature data, we started a research work to verify, in a large Italian prescription area (3,847,000 inhabitants), any differences between off-patent generic and off-patent branded drugs, with the aim of finding any differences in outcomes which could explain the current low use of off-patent generic drugs (15% with respect to quantity, and 7% of expenditure) [4]. The proposed study analyzed two groups of patients with metabolic, cardiovascular, psychiatric, and osteoporotic disease by determining a drug to be studied, with the purpose to assess any differences in compliance and persistence with treatment, mortality and more/less use of other health resources. Data on outcomes in real clinical practice in the two groups of patients was superimposable from the statistical point of view, and there were no differences in terms of average age and gender at enrolment. Data on compliance and persistence and on mortality appear to be similar in the two groups (off-patent generic vs. off-patent branded drugs); it is then necessary to add the potential resource saving for patients deriving from the larger use of off-patent generic drugs, including the co-payment of the costs of therapy by patients. Through the mechanism of the reference price, the price difference between off-patent branded drugs and off-patent generic drugs in Italy is completely charged on patients; in 2012, Italian Medicines Agency (AIFA) data [4] estimated a cost for citizens of about 1 billion euros a year due to the larger use of off-patent branded drugs than off-patent generic drugs.

However, this extra expenditure by the citizens due to the higher number of prescriptions of off-patent branded drugs instead of generics may not be a neutral phenomenon in the patients' clinical history. There is now consolidated evidence that adherence to therapy is a fundamental factor to assess the effectiveness of a treatment in patients treated for the prevention of cardiovascular risk [27]. Indeed, in the treatment of many chronic conditions such as hyperlipidemia or hypertension, there is a big gap between evidence-based recommendations and actual clinical practice [28], [29]. Several papers compared patients' adherence to the long-term incidence of acute cardiovascular events: only the group of highly-adherent patients reported a significantly lower risk of acute cardiovascular events with respect to the low-adherence group [30], [31], [32].

Studies conducted in the United States show quite clearly that the co-payment of drugs by the patients (called “ticket” in Italy) can contribute to a sub-optimal adherence to pharmacological therapies, up to their interruption. These papers [23], [24] analyzed adherence to therapy as a function of co-payment. According to Ellis et al. [23], if the co-payment share is fixed or equal to zero after two years of treatment, more than 70% of patients remain under treatment with statins, whereas monthly co-payments over 20 USD (14.60 EUR) reduce adherence to therapy to less than 30% of the patients originally treated with statins. The same applies to oral blood glucose lowering drugs, with which an increase of about 10 or 20 USD (about 7.30 or 14.60 EUR) in co-payment reduces the quantity of drugs taken daily by patients in a statistically significant way.

Together with these important observations, we need however to consider some limitations of this study. First of all, the clinical variables considered may not be complete to define the comparison sample: e.g., no consideration was given to familiarity, the patients' clinical history, and lifestyle elements that may influence the comparison. Patients' severity was only assessed by surrogate tools (hospitalizations, examinations, and use of other drugs). The period of observation, although sizeable (34 months), may not be sufficient to corroborate the outcome indicators considered. Furthermore, observational studies carried out using administrative databases have some limitations. The collected data directly come from invoicing by pharmacies; they give therefore a real estimate of prescribed and dispensed drugs, but not of the actual use of the drugs by patients. They also lack clinical data: since they are created for accounting purposes, they completely leave out data on the patients' lifestyle, on symptoms and diagnoses, and on intermediate outcome indicators (vital signs or biochemical levels) [33].

Patients actually analyzed for compliance and persistence were 16.90% of enrolled patients (No. 347,075), after applying the exclusion criteria in order to obtain two homogeneous comparison groups. In spite of these limitations, the study shows that the Italian NHS would benefit from relevant incremental resources, which could be destined to reimbursement or use of innovative therapies, if Italy got in line with the mean levels of generics use in advanced European countries. From the socio-economic point of view, off-patent generic drugs appear to be a very useful tool, allowing to obtain the same therapeutic effectiveness by improving the economic impact on patients and, in the end, on our National Health Service (NHS). A recent WHO document [19] mentions various examples of how to improve the efficiency of health systems, given the same employed resources, by eliminating waste and rationalizing inefficiency. The document identifies 10 key factors of inefficiency of health systems, indicating the insufficient use of off-patent generic drugs [19]; in the first position, off-patent generic drugs can bring about an increased efficiency in health systems and increase the percentage of population benefitting from a medical care plan. In the long term, indeed, introducing an increasing percentage of off-patent generic drugs in medical prescriptions involves direct and indirect savings for the NHS and the patients, which then makes more resources available to invest in research and innovation [34]. Off-patent generic drugs have a high social value, because they represent the main sustainable access to therapies for the most critical diseases in all socio-economic sectors, in the face of constantly increasing health requirements.
